# Assessment of Awareness and Perceptions of Healthcare Professional Graduates Regarding the Use of Clear Aligners in Orthodontic Treatment

**DOI:** 10.7759/cureus.46348

**Published:** 2023-10-02

**Authors:** Vinus Shivlani, Priyanka Niranjane, Priyanka Paul

**Affiliations:** 1 Public Health Dentistry, Sharad Pawar Dental College and Hospital, Datta Meghe Institute of Higher Education and Research (Deemed to be University), Wardha, IND; 2 Orthodontics, Sharad Pawar Dental College and Hospital, Datta Meghe Institute of Higher Education and Research (Deemed to be University), Wardha, IND

**Keywords:** orthodontics, awareness, health professionals, perception, clear aligners

## Abstract

Background

Clear aligners have been one of the most recent advancements in the field of orthodontics. With an increasing demand for aesthetics, the use of clear aligners has progressed, but knowledge is still lacking among the population. Therefore, this study aims to evaluate the perception and awareness of health professional graduates regarding the use of clear aligners in orthodontic treatment.

Materials and methods

A draft questionnaire was formulated and validated consisting of eight open-ended questions assessing the perception of respondents and two close-ended questions assessing awareness of clear aligners. A web-based survey was performed. The sample (n=438) population included graduates of different health professions including medical (248), Ayurveda (114), nursing (18) and physiotherapy(58). A Google form was prepared and the link was circulated to all. The response was sought in terms of Likert scales.

Results

A total of 438 graduates responded to the questionnaire, of which 56% were from medical faculty, 26% from Ayurveda, 13.20% from physiotherapy, and 4.1% from nursing. On average, the awareness and perception of health professional graduates regarding the use of clear aligners in orthodontics was average to low. Among all the health professions, the graduates from the medical faculty showed maximum knowledge regarding clear aligners, whereas the awareness amongst the nursing students was the least.

Conclusion

On assessing the knowledge of health professional graduates from different faculties regarding clear aligners, statistically different results were found for responses from all faculties. Since it is one of the most recent advancements in the field of orthodontics, there is a need to bridge this knowledge gap among health professionals, which would help them guide their patients to seek the best treatment modality at the appropriate age.

## Introduction

Clear aligners are a form of removable orthodontic appliance that helps in correcting different forms of malocclusion. Malocclusion is the third most common dental problem after caries and periodontal problems. It is considered as third most common dental problem which is affected by both genetic and environmental factors [1]. Malocclusion not only affects the physical appearance of a person but also affects the psychological behaviour and confidence of a person [2]. There are several treatment options to correct malocclusions which include removable orthodontic appliances, fixed orthodontic appliances, myo-functional appliances, and surgical orthodontics. These treatment procedures have several disadvantages including patient discomfort, pain, and masticatory and aesthetic problems [2]. To overcome these disadvantages, a newer treatment modality using clear aligners was introduced. With the increasing demand for aesthetics in dentistry, patients desire an aesthetically pleasing alternative to the traditional orthodontic treatment.

The concept of clear aligners was first introduced in dentistry by Kesling in the year 1945 [3]. Kesling proposed the use of clear aligners as positioners to refine the final orthodontic treatment. In 1998, Align Technology (Santa Clara, USA) introduced a system called Invisalign. This is considered to be an aesthetic alternative to conventional orthodontic treatment which can be used for correction of mild to moderate cases. Later, auxiliary tools integrated with clear aligner treatment were introduced which improved its clinical efficiency, stability and treatment outcome [4]. With the evolution of graphic designing, it was integrated with digital diagnostic tools, programmed virtual treatment plans, and biomechanical designing. A three-dimensional visual interface that allows dentists to customize the treatment plan and monitor the progress to incorporate the modifications as per requirement [5].

The material used in the preparation of clear aligners is polyurethane plastics. The material is transparent hence it provides excellent aesthetics and, as the aligners are removable, they provide adequate oral health maintenance [6]. Although clear aligners were introduced years back, but they did not gain much popularity in those decades because of a lack of awareness and promotion. With the advances in technology and promotion techniques like social media and digital marketing, the use of clear aligners in orthodontics has gained immense popularity in the last decade [6]. With advancements in the field of biomechanics, the use of clear aligners has evolved drastically and can be used for various malocclusions. However, even though these clear aligners provide several advantages to patients, their efficacy is still debated in the literature [7]. There is a need to increase awareness regarding the use of clear aligners which would enhance its popularity among people who want to seek orthodontic treatment. Spreading awareness improves the state of mind and consciousness of the people, which in turn will help in enhancing oral health [8]. In 2022, a study was performed in Saudi Arabia assessing the knowledge of dentists regarding clear aligners which showed moderate awareness [9] and in 2022 similar study was performed in Gujarat, India to assess knowledge among dentists which showed a high level of awareness among them [10]. As assessed by a study in Saudi Arabia in 2022, the awareness amongst the general public was also very low [11].

Although many studies have been undertaken to assess the awareness of clear aligners amongst dentists, patients undergoing orthodontic treatment, and the general population, the perception among health professionals is unexplored.

## Materials and methods

Type of study

A cross-sectional questionnaire-based study was performed. The study was reviewed and approved by the Institutional Ethics Committee of Datta Meghe Institute of Higher Education and Research (DMIHER). Healthcare professional graduates from different faculties like medical (n=250), ayurveda (n=100), nursing (n=50), and physiotherapy (n=100) that existed in the DMIHER campus were invited to participate in the study. The total number of graduates from all the institutes was around 500.

Sample size estimation was done with the non-probabilistic sampling method​​​​​​ using the formula,

n = Z^2^_1-α/2_ x p(1-p) / d^2^

Alpha (α) = 0.05

Estimated proportion (p) = 0.50

Estimated error (d) = 0.05

The minimum sample size needed came to n=385. Accordingly, the sample distribution was medical (n=220), Ayurveda (n=80), physiotherapy (n=65), and nursing (n=20). The draft questionnaire was formulated consisting of 10 questions, out of which eight were close-ended, assessing the perception of the participants, and two were open-ended, assessing awareness regarding clear aligners and their choice of treatment. A Google form was created and the link was circulated to all the participants. The responses were sought on the basis of a 5-point Likert Scale where 1=Strongly agree, 2=Agree, 3=Neutral, 4=Disagree, and 5=Strongly disagree.

Study questions

Question Concerning the Participant Information

It included the demographic details of participants and the healthcare profession faculty to which they belong (Question 1).

Question Concerning Awareness of Participants

It was assessed as an open-ended question on whether they knew about this treatment and their source of knowledge regarding this (Question 2).

Questions Concerning Their Perception

This question assessed the costing, conditions treated with aligners, and the efficiency of aligners compared to conventional treatment (Questions 3-9).

Questions Concerning Choice of Treatment

This question assessed the choice of participants choosing clear aligners for orthodontic treatment (Question 10).

The full questionnaire is available in the Appendices.

Statistical analysis

SPSS Statistic software (IBM Corp., Armonk, NY) was used to analyze the results. Descriptive statistics were used and the Chi-square test was performed to evaluate the association between the response of graduates and their awareness and perception regarding clear aligners used for orthodontic treatment. The response rate was 87.6%.

## Results

**Figure 1:**
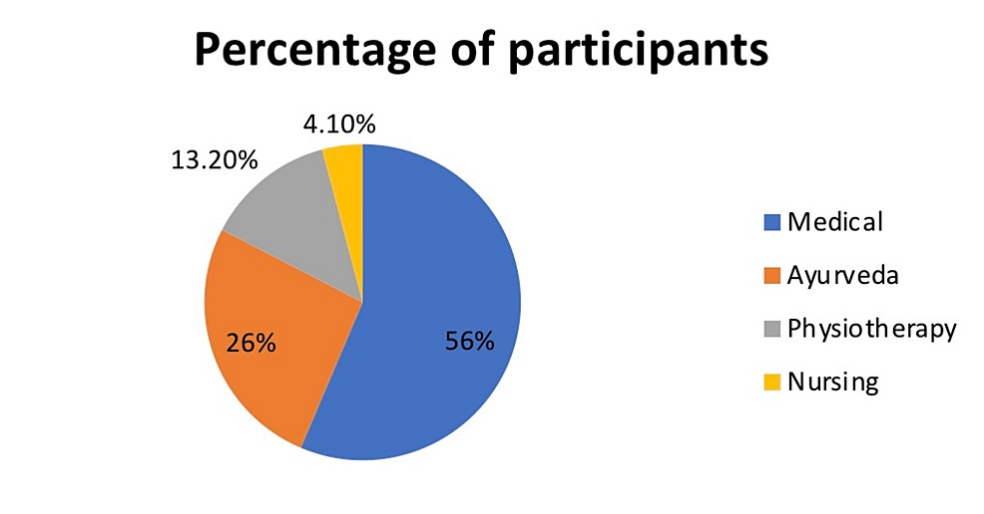
Percentage of respondents from different faculties Created by authors

The sample included 438 students from different faculties of the health profession. Out of this, 248 (56%) were graduates from the medical faculty, 114 (26%) from the Ayurveda faculty, 58 (26%) from physiotherapy, and 18 (4.10%) students from nursing (Figure 1).

Assessment of the questionnaire

**Figure 2:**
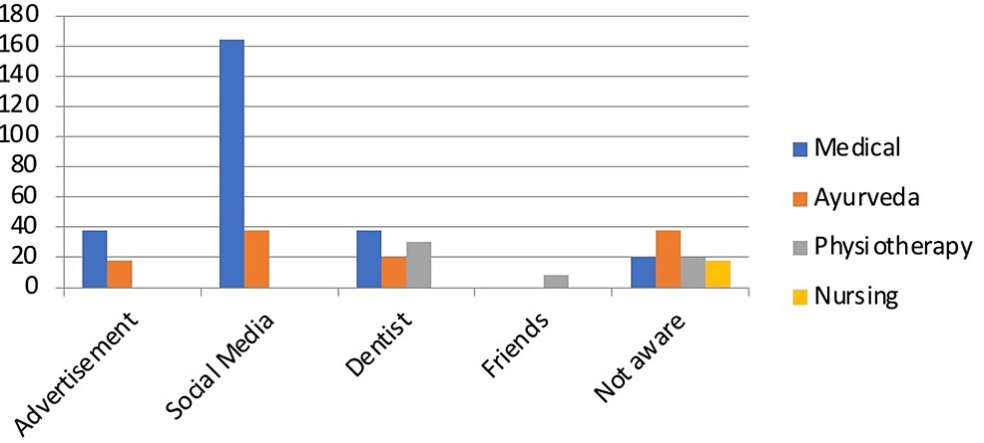
Response of participants on their source of knowledge on clear aligners (Question 2) Created by author

The responses were collected on the basis of a Likert scale. Among the respondents, 342 students were aware of clear aligners used as an orthodontic treatment modality. The rest 96 respondents were unaware of the use of clear aligners in orthodontics. The maximum of aware respondents were from the medical faculty (88%), followed by Ayurveda (66.6%), physiotherapy (65%), and the least from nursing (0%). The source of their knowledge regarding clear aligners was mentioned as the advertisement, social media, friends, fellow colleagues, and their dentists. The majority of them had heard about clear aligners from social media (Figure 2).

Among the graduates of all the professions, the medical students depicted maximum positive response (91%) regarding their awareness about clear aligners, whereas the awareness was less among Ayurveda (66%) and physiotherapy students ( 65.5%) and the least amongst the students of nursing (0%) (Table 1).

**Table 1:** Response of participants from different faculties

			Response					
Question no.	Question	Faculty	Strongly agree	Agree	Neutral	Disagree	Strongly disagree	p-Value
Q1.	I am aware of clear aligners used in orthodontic therapy.	A) Medical; B) Ayurveda; C) Physiotherapy; D) Nursing	A) 96 B) 38 C) 12 D) 0	A) 122 B) 36 C) 20 D) 0	A) 20 B) 40 C) 24 D) 0	A) 10 B) 0 C) 0 D) 18	A) 0 B) 0 C) 0 D) 0	0.001
Q2.	Clear aligners are better than wires and braces	A) Medical; B) Ayurveda; C) Physiotherapy; D) Nursing	A) 58 B) 18 C) 18 D) 0	A) 136 B) 20 C) 20 D) 0	A) 54 B) 18 C) 20 D) 18	A) 0 B) 58 C) 0 D) 0	A) 0 B) 0 C) 0 D) 0	0.001
Q3.	Clear aligners can treat all types of orthodontic problems	A) Medical; B) Ayurveda; C) Physiotherapy; D) Nursing	A) 20 B) 0 C) 18 D) 0	A) 134 B) 18 C) 0 D) 0	A) 76 B) 38 C) 40 D) 18	A) 18 B) 58 C) 0 D) 0	A) 0 B) 0 C) 0 D) 0	0.001
Q4.	Clear aligners must be changed after every 2-3 months	A) Medical; B) Ayurveda; C) Physiotherapy; D) Nursing	A) 58 B) 0 C) 18 D) 0	A) 152 B) 56 C) 0 D) 0	A) 18 B) 18 C) 40 D) 18	A) 0 B) 40 C) 0 D) 0	A) 0 B) 0 C) 0 D) 0	0.001
Q5.	Clear aligners are completely invisible	A) Medical; B) Ayurveda; C) Physiotherapy; D) Nursing	A) 38 B) 38 C) 18 D) 0	A) 136 B) 136 C) 0 D) 18	A) 54 B) 18 C) 40 D) 0	A) 20 B) 78 C) 0 D) 0	A) 0 B) 0 C) 0 D) 0	0.001
Q6	Clear aligners can limit the type of food you can eat	A) Medical; B) Ayurveda; C) Physiotherapy; D) Nursing	A) 30 B) 34 C) 2 D) 0	A) 152 B) 6 C) 5 D) 0	A) 62 B) 51 C) 36 D) 18	A) 3 B) 5 C) 4 D) 0	A) 1 B) 22 C) 0 D) 0	0.001
Q7	Clear aligners are effective in treatment of crooked teeth	A) Medical; B) Ayurveda; C) Physiotherapy; D) Nursing	A) 145 B) 6 C) 10 D) 2	A) 30 B) 16 C) 8 D) 2	A) 60 B) 56 C) 32 D) 14	A) 13 B) 20 C) 8 D) 0	A) 0 B) 16 C) 0 D) 0	0.001
Q8	Cost of clear aligners is very high	A) Medical; B) Ayurveda; C) Physiotherapy; D) Nursing	A) 154 B) 28 C) 8 D) 0	A) 68 B) 18 C) 4 D) 2	A) 24 B) 38 C) 35 D) 14	A) 4 B) 4 C) 9 D) 2	A) 0 B) 26 C) 2 D) 0	0.001

**Figure 3:**
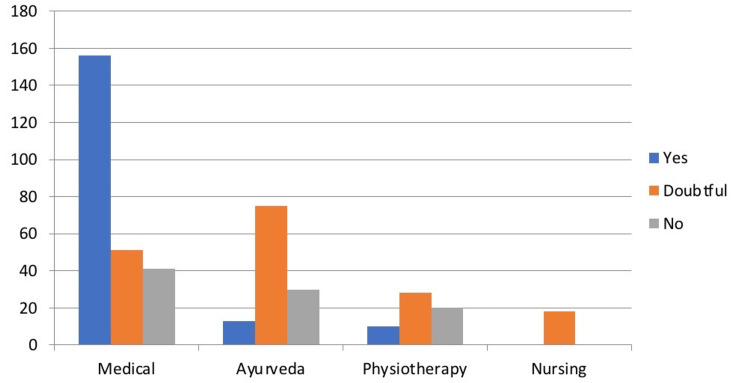
Response of participants on their choice of clear aligners as a treatment option (Question 10) Created by author

Responding to the statement regarding whether the efficiency of clear aligners is better than conventional orthodontic treatment, 23.4% of medical students strongly agreed with this statement and 21.7% of students gave a neutral response to the statement. Among Ayurveda students, 15.7% students strongly agreed while 50.8% disagreed with this statement. Among physiotherapy students, 31% students agreed while 34.4% gave a neutral response. All of the nursing students gave a neutral response to this statement. In the response concerning whether clear aligners can treat all types of orthodontic problems, the majority of medical graduates (54%) strongly agreed to this statement, the majority of Ayurveda (50.8%) students disagreed, 68.9% of physiotherapy students gave a neutral response to this statement. 61.3% of medical and 49% of Ayurveda graduates agreed that the clear aligners must be changed every 2-3 weeks whereas, 68.8% of physiotherapy graduates gave a neutral response to this statement (Table 1). Moreover, 54.8% of medical graduates agreed that clear aligners are completely invisible. On the contrary, 68.4% of Ayurveda students disagreed and 68% of physiotherapy students gave a neutral response to this (Table 1). A maximum of medical students agreed to the statement that clear aligners can limit the type of food we can eat as well as the cost of clear aligners is very high. Contrary to this, a maximum of nursing, Ayurveda, and physiotherapy students gave a neutral response to this question (Table 1). The majority of medical students preferred aligners as a treatment option while others remain doubtful of this (Figure 3).

The p-value for all the statements was significant, indicating that the differences in perception of graduates from different faculties were significant.

## Discussion

Malocclusion has a severe impact not only on the oral health of the child but also on physical appearance and has psychological impact [12]. The knowledge and awareness of the general population regarding malocclusion play an important role in the early detection and proper management of the same [13]. To the knowledge of the authors, there is no evidence of studies comparing the group of population that includes graduates from medical, Ayurveda, physiotherapy and nursing. The awareness amongst these healthcare graduates is essential as they are the healthcare providers who could guide the patients to opt for the best treatment modality. Adding to this, health professionals must also be aware of the malocclusion and its possible complications to guide their patients to seek the appropriate treatment at the proper age.

The literature shows evidence of several studies assessing awareness among dentists, dental graduates, the general public, and orthodontists; these include a study conducted in Saudi Arabia in 2022 assessing the dentist’s knowledge regarding the use of clear aligners which showed moderate awareness among the dentists and major part of respondents did not opt for this modality of treatment for their patients [9]. A study performed in 2022 in Gujarat, India assessed the awareness of dental students regarding clear aligners which concluded that 93.5% of dental interns and 83.6% of dental undergraduates were aware [10]. A study performed in 2022 in Saudi Arabia assessed the knowledge among the general public, in which out of 934 respondents, only 19.6% were aware of clear aligners [11]. A survey was performed in 2020 to assess the awareness and perception among orthodontists and other dental specialities, which concluded that 93.5% of orthodontists and 60% of general dentists and dentists of other specialties are aware of clear aligners [14]. Another study performed at Malaysian Medical University, which compared the awareness of clear aligners amongst medical and dental students, concluded a significant difference in awareness (p=0.002), where dental students have a higher knowledge than medical, and the reason for this could be the exposure through their curriculum [15].

In our survey comparing the efficacy of clear aligners with conventional treatment, 40.2% of total respondents strongly agreed that clear aligners are better, which was similar to the study of Saudi Arabia [11] that assessed the perception regarding the effectiveness of clear aligners where the majority of people (30.6%) agreed that it is better than conventional treatment modality. One of the major advantages of clear aligners over conventional orthodontic treatment is the aesthetic property which enhances its use for adult orthodontics. Assessing this, our survey depicted the maximum positive response (44.7%) for the perception of respondents regarding the invisibility of clear aligners, which was similar to a study performed in Saudi Arabia that depicted that the majority of participants (33.9%) agreed and 31% strongly agreed that clear aligners are aesthetically superior to conventional treatment [11].

According to a study, nowadays parents are willing to spend money for their children in order to achieve better aesthetics and therefore they are willing to pay more in order to get the best orthodontic treatment [16]. On assessing the efficiency of clear aligners in treating all types of malocclusion, our study showed the majority (43.4%) of respondents gave a neutral response regarding the use of clear aligners for all types of orthodontic problems which depicts a knowledge gap regarding the efficiency of clear aligners. In literature, there are certain studies performed which suggest that clear aligners must not be used for difficult orthodontic cases [17] which is still a matter of controversy. Assessing the response for time duration for changing aligners from our survey depicts that the majority of respondents (36.5%) were unsure regarding the duration of wearing aligners and only 14.6% of respondents felt that aligners must be changed timely which was similar to the data from the literature that recommended the wearing duration of clear aligners as 2 weeks [18].

From our study, only 11.9% of respondents disagreed with the statement that clear aligners limit the type of food that the patients can eat, which was similar to the survey of Saudi Arabia assessing the inconveniences faced by the general public that shows that 27.9% of respondents agreed that clear aligners cause less discomfort than conventional orthodontic treatment [11]. However, this was contradictory to the results of a study from the Malaysian University where 43.6% of medical and dental students were not sure if the clear aligners would create problems in mastication [15]. The cost of clear aligners still remains a major issue which limits its use in orthodontics. Assessing this, in our survey 89% of medical graduates, 40.35% of Ayurveda, 20% of physiotherapy, and 1.12% of nursing graduates agreed with the statement that the cost of clear aligners is very high (Table 1), and in total, 31.1% respondents were unaware of its cost; in comparison, the study performed in Malaysian Medical University shows that majority (57%) of medical and dental graduates felt that the cost of clear aligners is very high [15].

The last question was directed towards assessing the choice of whether the respondents would opt for clear aligners, which showed that the majority (42.9%) would consider clear aligner if required, and among these, the maximum respondents belong to the medical faculty (62%) while the choice for maximum respondents from Ayurveda, physiotherapy, and nursing still remains doubtful. To the best of our knowledge, this result cannot be compared to other studies as the literature does not show evidence of any study comparing the choice of treatment if required by the different disciplines of healthcare graduates.

There were certain limitations of this study. It was a cross-sectional study. Since the survey link was circulated through Google form, the difficulties faced by the respondents while filling out the form could not be assessed. The study assessed perceptions of health science in this region, hence caution must be exercised while generalizing the results in relation to other contexts. Further studies could be performed to widen the sample and include health science graduates from other faculties as well.

## Conclusions

Clear aligners can be considered an aesthetic alternative to conventional orthodontic treatment. On assessing the knowledge of healthcare professional graduates from different faculties regarding clear aligners, the awareness among medical graduates was moderate, moderate to low among Ayurveda and physiotherapy graduates, and least among nursing graduates. There was a significant difference in the perception of graduates regarding clear aligners. Since it is one of the most recent advancements in the field of orthodontics, there is a need to bridge this knowledge gap among health professionals, which would help them guide their patients in seeking the best treatment modality at the appropriate age.

## Appendices

Questionnaire

Email

Question 1 - Which faculty do you belong?

Medical

Ayurveda

Physiotherapy

Nursing

Question 2 - Have you heard about clear aligners? If yes from whom?

Question 3 - I am aware of clear aligners used in Orthodontic teeth alignment

Strongly agree

Agree

Neutral

Disagree

Strongly Disagree

Question 4 - Clear Aligners are better than wires and braces.

Strongly agree

Agree

Neutral

Disagree

Strongly disagree

Question 5 - Clear Aligners can treat all types of orthodontic problems.

Strongly agree

Agree

Neutral

Disagree

Strongly Disagree

Question 6 - There will be a need to change clear aligners every 2-3 weeks.

Strongly agree

Agree

Neutral

Disagree

Strongly disagree

Question 7 - Clear aligners are completely invisible.

Strongly agree

Agree

Neutral

Disagree

Strongly Disagree

Question 8 - Clear Aligners can limit the type of food you can eat.

Strongly agree

Agree

Neutral

Disagree

Strongly Disagree

Question 9 - Clear aligners are effective in treatment of crooked teeth.

Strongly agree

Agree

Neutral

Disagree

Strongly Disagree

Question 10 - The cost of clear aligners is very high.

Strongly agree

Agree

Neutral

Disagree

Strongly disagree
